# Kbus/Idr, a mutant mouse strain with skeletal abnormalities and hypophosphatemia: Identification as an allele of 'Hyp'

**DOI:** 10.1186/1423-0127-18-60

**Published:** 2011-08-20

**Authors:** Kenji Moriyama, Atsuko Hanai, Kazuyuki Mekada, Atsushi Yoshiki, Katsueki Ogiwara, Atsushi Kimura, Takayuki Takahashi

**Affiliations:** 1 Department of Medicine & Clinical Science, School of Pharmacy and Pharmaceutical Sciences, Mukogawa Women's University, Nishinomiya 663-8179, Japan; 2 Department of Developmental Biology, Institute for Developmental Research, Aichi Human Service Center, Kasugai 487-0392, Japan; 3 Division of Experimental Animal Research, BioResource Center, RIKEN Tsukuba Institute, Tsukuba 305-0074, Japan; 4 Laboratory of Reproductive and Developmental Biology, Faculty of Science, Hokkaido University, Sapporo 060-0810, Japan

**Keywords:** Bone defects, Hypophosphatemia, Mouse model, Phex, Hyp, XLH

## Abstract

**Background:**

The endopeptidase encoded by *Phex *(phosphate-regulating gene with homologies to endopeptidases linked to the X chromosome) is critical for regulation of bone matrix mineralization and phosphate homeostasis. PHEX has been identified from analyses of human X-linked hypophosphatemic rickets and Hyp mutant mouse models. We here demonstrated a newly established dwarfism-like Kbus/Idr mouse line to be a novel Hyp model.

**Methods:**

Histopathological and X-ray examination with cross experiments were performed to characterize Kbus/Idr. RT-PCR-based and exon-directed PCR screening performed to identify the presence of genetic alteration. Biochemical assays were also performed to evaluate activity of alkaline phosphatase.

**Results:**

Kbus/Idr, characterized by bone mineralization defects, was found to be inherited in an X chromosome-linked dominant manner. RT-PCR experiments showed that a novel mutation spanning exon 16 and 18 causing hypophosphatemic rickets. Alkaline phosphatase activity, as an osteoblast marker, demonstrated raised levels in the bone marrow of Kbus/Idr independent of the age.

**Conclusions:**

Kbus mice should serve as a useful research tool exploring molecular mechanisms underlying aberrant *Phex*-associated pathophysiological phenomena.

## Background

During the maintenance of the KYF/MsIdr strain of mouse, which earlier spontaneously yielded an abnormal behavior-displaying Usher-1D model, BUS/Idr [1,2], we recognized the occurrence of dwarfism-like short-tailed individuals, which displayed distinct bustling behavior. We have established the mutant as a new strain, Kbus/Idr, through brother-sister mating, and attempted to specify the responsible gene(s), as dealt with in this paper.

## Introduction

Osteogenesis is controlled by osteoblast/osteoclast functional balance in close association with phosphate (Pi) homeostasis regulated by complicated systems operating across the parathyroid gland, intestine, bone and kidney [3,4]. Parathyroid hormone (PTH), 1,25-vitamin D_3 _and calcium-sensing receptors constitute the classic pathway of Pi/calcium homeostasis, which is essential for bone differentiation and remodeling. In addition, two important key mediators have been identified through clinical observation and subsequent molecular approaches, fibroblast growth factor-23 (FGF23) and a phosphate-regulating gene product with homology to endopeptidases linked to the X chromosome (PHEX). Gain-of-function mutations of *FGF23 *lead to autosomal dominant hypophosphatemia/osteomalacia [5], while its loss-of-function mutations are causative of recessive familial tumoral calcinosis with hyperphosphatemia [6,7]. FGF23, secreted mainly from osteoblasts/osteocytes [8,9], is a potent inhibitor of renal Pi reabsorption, leading to phosphate wasting. This phosphaturic hormone binds to renal FGF23 receptor (FGF23r)/Klotho heterodimeric molecules much more tightly than to FGF23r alone, thereby exerting marked inhibitory actions against renal Pi reabsorption [10,11].

PHEX, another potent mediator of phosphate homeostasis, has been identified from analyses of human X-linked hypophosphatemic rickets (XLH) [12] and Hyp mutant models [13,14]. Loss-of-function mutations of *PHEX/Phex *lead to skeletal abnormalities and hypophosphatemia, and are genetically fully dominant [15]. Aberrant *PHEX/Phex *expression also results in abnormalities in cartilages [16,17] and teeth [18]. Phex belongs to the M13-type plasma membrane-integrated metalloendopeptidase family, and is expressed exclusively in cells of the osteoblast/osteocyte lineage [19,20]. Accumulating evidence indicates that the Phex substrates are protease-resistant acidic serine-aspartate-rich motif peptides (ASARM peptides) generated from small integrin-binding ligand, N-linked glycoproteins (SIBLING proteins) by cathepsin actions [21-29]. Phex interacts with and degrades ASARM peptides of SIBLINGs, such as matrix extracellular phosphoglycoprotein (MEPE), osteopontin and dentin matrix protein 1. Although the SIBLINGs are not highly homologous in structure [30], their ASARM peptides bind, in a phosphorylation-dependent manner, to matrix Ca × PO_4 _to inhibit mineralization. Both SIBLINGs and ASARM peptides are increased in Hyp and human XLH and strongly inhibit renal Pi reabsorption [23,31]. Finally, transgenic mice overexpressing MEPE in bone mimic the Hyp model, displaying growth and mineralization defects with altered bone-renal vascularization [32].

To date, six Phex mutant models, Hyp (a 3'-deletion of the *Phex *gene) [13,14], Gy (partial deletion of both *spermine synthase *and *Phex*) [13,33], Phex(Ska1) (skipping of exon 8) [34], Hyp-J2 (deletion of exon 15) [35], Hyp-Duk (deletion of exons 13 and 14) [35], and Phex(pug) (glycosylation defects due to Phe-to-Ser substitution at a.a. 80 of Phex) [36] have been reported, while over 260 human disease-associated *PHEX *mutations have been identified [37-41]http://http:/www.phexdb.mcgill.ca. We have now established a dwarfism-like strain of short-tailed mouse, Kbus/Idr, carrying a novel intragenic deletion of the *Phex *gene.

## Materials and methods

### Mice

Highly inbred Kbus/Idr mice, maintained for over 20 generations were used. Kbus mice were of KYF/MsIdr-origin, and hence KYF mice were used as control animals. All were housed in an air-conditioned room in the Institute for Developmental Research, Aichi Human Service Center, Kasugai, Japan, with a constant temperature (23 ± 1°C) and humidity (55 ± 10%) on a 12:12-hr light-dark cycle with lights on at 7:00 a.m., with free access to standard laboratory diet (CE-2; CLEA Japan Inc.). The animals were handled in accordance with the institute guidelines. Frozen Kbus embryos are now available from RIKEN BioResource Center, Tsukuba, Japan.

### Histopathology and X-ray examination

The detailed methods for histopathological examination are described in the legends of Additional file 1, Figure S1 and Additional file 2, Figure S2. To evaluate bone density, age-matched Kbus and KYF mice were subjected to X-ray irradiation at 40 kV and 100 mA for 6 seconds, together with eleven ceramid strips with gradual X-ray transmissivity rates (X-TR), 1 to 11, where X-TR 11 represented the highest TR.

### Biochemical experiments

Blood was collected from deeply anesthetized mice, 8 weeks of age, and mixed with an equal volume of 10% trichloroacetic acid and centrifuged at 12,000 × g for 10 min. The supernatants obtained were directly subjected to Pi assay, according to the method of Fiske and SubbaRow [42]. Bone marrow stromal cells (BMSCs) were obtained from the femurs, washed once with phosphate-buffered saline (PBS) and sonicated in 50 mM Tris-1 mM EDTA, pH 6.8, containing 0.1% Triton X-100. Alkaline phosphatase (ALP: osteoblast marker) and tartrate-resistant acid phosphatase (TRAP: osteoclast marker) were assayed at 37°C in 0.1 M glycine-NaOH, pH 10.0, and 0.1 M acetate buffer containing 0.3 M sodium tartrate, pH 4.2, respectively, using 2 mM nitrophenylphosphate (NPP) as the substrate. One unit of enzyme was defined as that releasing 1 μmole of Pi from NPP per min. Protein was measured with a BCA Protein Assay Reagent Kit (Pierce). Serum ALP levels were assessed as described above with x2 diluted serum samples from 14-week-old males. The results were presented as the means ± SEM, and statistically analyzed by Student's *t*-test, where differences of *P *< 0.05 were considered significant.

### Cross experiments and PCR analysis

The phenotype of all progeny was judged at 8-weeks of age, based on the tail length and dwarfism-like appearance, as well as bustling behavior. The results of Kbus-KYF crosses gave simple segregation patterns in these traits, as shown in the Results section.

For RT-PCR-based screening for mutations of mouse *Phex*, total RNA fractions were prepared from bony tissues of 3 to 12-day-old mice (Total RNA purification kit; Stratagene). Oligo *dT*-primed and *Phex*-specific reverse R6 (Additional file 3, Table S1)-primed first strand cDNAs were prepared using Superscript III (Stratagene), and PCR was carried out with AmpliTaq-Gold (Roche-Applied Biosystems). DNAs were prepared by digestion of homogenized fresh livers with proteinase K in the presence of pancreatic RNase, EDTA and SDS, followed by extraction with phenol. The primer sets used for RT-PCR-based and exon-directed PCR-based screening are shown in Additional file 3, Table S1. The nucleotide sequences of all PCR products were verified with an ABI PRISM 310 genetic analyzer (Applied Biosystems).

## Results

### Characterization of Kbus/Idr mice

Kbus mice were distinguishable from KYF mice because of a growth defect resulting in a dwarfism-like appearance and short tail (Additional file 1, Figure S1). They exhibited bustling behavior, but swam well, indicating vestibular function to be normal. Kbus mice also exhibited normal startle responses to sounds (popping with hands), indicating that they heard. Since no degenerative features of Corti's organ or spiral ganglion cells were observed in Kbus inner ears (not shown), we concluded that they had no serious inner ear defects. We further noted that the bustling behavior in Kbus was absolutely linked to bone defects in Kbus-KYF crosses, but was almost nullified in F2 progeny of Kbus-C57BL/6 crosses, suggesting the behavioral abnormality to be dependent on the KYF background. Therefore, our primary attention in this study was focused on skeletal abnormalities in Kbus mice.

**Figure 1:**
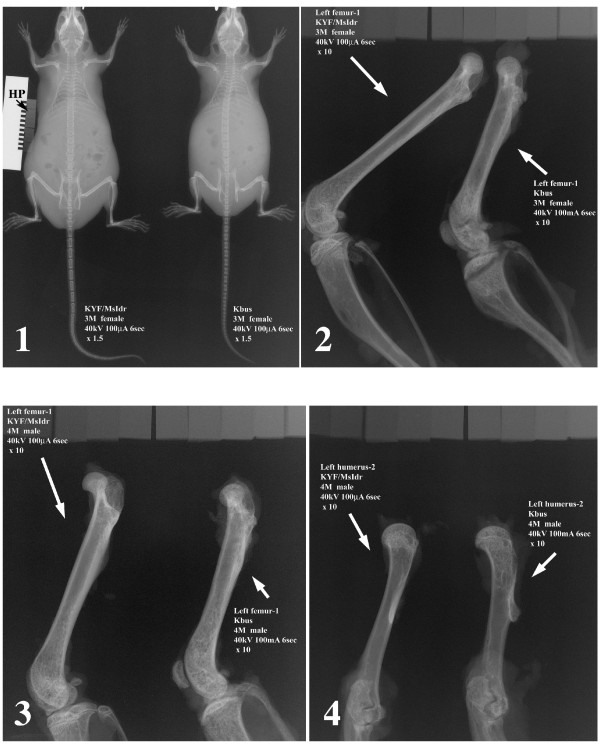
**X-ray examination for assessing skeletal deformation and mineralization defects in Kbus mice**. X-rays were applied at 40 kV and 100 mA for 6 seconds to Kbus and KYF adult (panel 1) femurs (panels 2 and 3) and humeri (panel 4). A set of eleven hydroxyapatite plates with different X-ray transmissivity rates (X-TR), X-TR1 to 11, where 1 had the least X-TR, was used to assess bone mineralization. The hydroxyapatite plates (HP in panel 1) are seen in each panel (on the top in panels 2-4). For the cortical region of the central part of the tibia, X-TR values of 8~10 and 4~5 were obtained for Kbus and KYF, respectively.

Defective features in Kbus bones were apparent in cleared whole body skeletal preparations (Additional file 2, Figure S2) and histopathological examination (Additional file 4, Figure S3): Each of the long bones and bony segments of the tail was shorter than the couterpart in KYF mice, resulting in a shorter tail and dwarfism-like features of Kbus mice. In Kbus cortical bones many sinuses were observed even at 12 weeks of age; consequently, the long bones of Kbus adults had a deranged Haversian system and branched marrows, indicating defective vascularization and incomplete differentiation of dense cortical bone. In Kbus cartilage, abnormally thick toluidine blue-positive growth plates were evident. Finally, the X-ray examinations revealed reduced bone density (Figure 1), X-TR values for the cortical bone of the central tibia being 8~10 for Kbus and X-TR 4~5 for KYF.

### Identification of Kbus mice linkage to a Hyp allele

In crosses between Kbus females and KYF males, all F1 progeny exhibited skeletal abnormalities, while only F1 females were affected in crosses of KYF females and Kbus males (Table 1). This, together with the backcross data, was consistent with the view that the Kbus phenotype was controlled by a dominant gene linked to the X chromosome. Phenotypic segregation in F2 males, but not females, was different from the theoretical values (double asterisks in Table 1), due most probably to the small number of F2 progeny.

**Table 1:** Phenotype segregation in cross experiments

*F1 progeny of Kbus × KYF *				
	normal	abnormal*		

female	0	15		

male	0	8		

***F2 progeny of F1(ab^1^) × F1(ab)***			***Backcross of F1(ab) × KYF***	

	normal	abnormal	normal	abnormal

female	0	7	6	6

male	2**	9**	6	9

***F1 progeny of KYF × Kbus***				

	normal	abnormal		

female	0	17		

male	14	0		

***F2 progeny of F1(ab) × F1(n^1^)***			***Backcross of F1(ab) × Kbus***	

	normal	abnormal	normal	abnormal

female	4	4	0	6

male	3**	12**	6	7

^1 ^ab and n, abnormal and normal in phenotype, respectively.

* Individuals displaying both short tail (body > tail in length) and bustling behavior were taken as abnormal.

** Significantly different from the theoretical values, assuming that the traits are inherited in an X-linked dominant manner.

**Figure 2:**
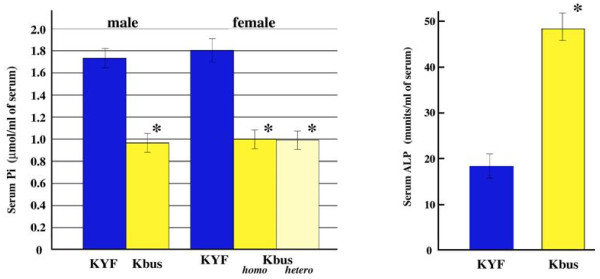
**Blood phosphorus levels in 8-week-old KYF and Kbus mice, showing Kbus mice to be hypophosphatemic (right panel)**. No significant variation was observed between homozygous and heterozygous Kbus females, indicating the hypophosphatemic character to be fully dominant. n = 3. Also refer to Additional file 3, Table S2. Serum ALP levels in 14-week-old KYF and Kbus mice, showing extremely high activities in Kbus (left panel). n = 5. Asterisks indicate the statistical significant difference for the values of age-matched KYF samples (*p *< 0.01).

**Figure 3:**
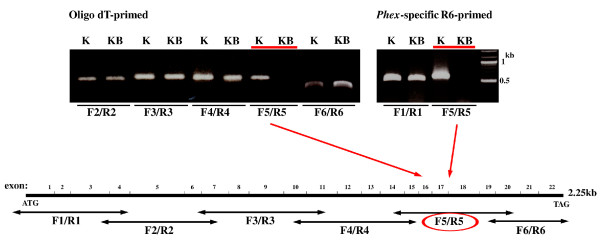
**RT-PCR screening of the *Phex *transcripts in Kbus bony tissues**. The primer sets used are shown in Additional file 3, Table S1. Oligo-*dT*-primed (left panel) and *Phex*-specific R6-primed (right panel) first strand cDNAs were prepared from Kbus (KB) and KYF (K) bony tissues. No PCR products were obtained for exon 16-18-associated regions, even with R6-primed cDNAs used as templates. The regions covered by different forward (F)/reverse (R) primers are indicated at the bottom. For further details, see the text.

**Figure 4:**
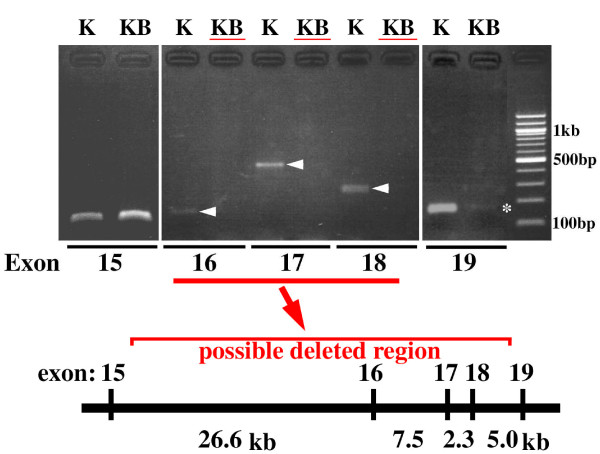
**Exon-directed PCR screening of the Kbus *Phex *gene**. The primer sets used are shown in Additional file 3, Table S1. With Kbus DNA, no PCR products were obtained with exons 16, 17 and 18, indicating an intragenic 10-40 kb deletion, as depicted at the bottom. For further details, see the text.

Given that Kbus mice displayed a bone matrix mineralization defect inherited in an X chromosome-linked dominant manner, we considered that the mutant might carry a Hyp allele. Indeed, blood Pi levels in Kbus were significantly low, and hypophosphatemic traits were also dominant (Additional file 3, Table S2 and right panel of Figure 2). In addition, serum ALP levels in Kbus were significantly higher than those of KYF values (48.5 ± 4.1 munits/ml of serum *vs*. 18.4 ± 2.7 munits/ml of serum, *p *< 0.01, n = 5; left panel of Figure 2), consistent with the data for Hyp and XLH [3]. We therefore attempted to detect mutations of the *Phex *gene. In RT-PCR experiments, we could obtain no PCR products for an exons 16-18-associated region (Figure 3). We emphasize here that we applied different sets of primers (not listed in Additional file 3, Table S1) to detect the region, and no set gave positive results, even when specific R6-primed first strand cDNAs were employed as templates (Figure 3). Although F6/R6-PCR products were regularly fewer in Kbus than in KYF in repeated experiments, the nucleotide sequences could be verified as essentially the same. Finally, no PCR products for exons 16, 17 and 18 were detected in the Kbus genome, indicating an intragenic deletion spanning 10-40 kb (Figure 4). Again, the PCR products for exon 19 were regularly fewer in Kbus than in KYF, but without nucleotide changes.

### High alkaline phosphatase activity in isolated Kbus BMSCs

In view of the aberrant *Phex*-induced reduction in skeletogenesis, the mutant elevated serum ALP activity appeared of interest, given that ALP was an osteoblast marker, and because Hyp BMSCs indicated reduced osteoblastogenesis and skeletogenesis in culture [24-26]. Examination of ALP and TRAP levels in total BMSCs demonstrated raised levels of only the former in Kbus as compared to KYF BMSCs independent of the age (Table 2). ALP-positive cells in the bone marrow could thus have been the source of high serum ALP.

**Table 2:** Alkaline phosphatase (AP) and tartrate-resistant acid phosphatase (TRAP) activities in isolated KYF and Kbus BMSCs

		AP activity	TRAP activity
4 wks	KYF (n = 6)	19.7 ± 1.8	61.2 ± 1.8

	Kbus (n = 6)	41.7 ± 4.1*	69.4 ± 4.0

8 wks	KYF (n = 7)	16.9 ± 1.9	58.6 ± 2.2

	Kbus (n = 8)	20.6 ± 3.7	52.8 ± 3.4

16 wks	KYF (n = 8)	8.7 ± 0.7	46.9 ± 2.0

	Kbus (n = 8)	18.7 ± 2.9*	43.5 ± 2.3

32 wks	KYF (n = 6)	7.3 ± 0.3	43.0 ± 3.2

	Kbus (n = 6)	14.1 ± 2.7*	45.4 ± 3.6

munits/mg of protein (mean ± SEM)

Since no significant differences were found between male and female samples, data from both sexes were combined.

*, Significantly different from the values of age-matched KYF samples (*P *< 0.05).

## Discussion

The present study demonstrated the Kbus strain, here referred to as Hyp(Kbus), to carry a novel genetic alteration of the *Phex *gene. It was suspected that the distinct bustling behavior and skeletal abnormalities might be controlled by a single gene. Abnormal behavior, such as circling and head tossing and tilting, is a typical sign of inner ear defects in mice [1,2]; and two Hyp models, Gy and Hyp-Duk [34,36], have also been reported to display abnormal behavior. However, deafness and endolymphatic hydrops due to the *Phex^Hyp-Duk ^*mutation exhibit background-dependent variable expression [35,43]; and in the Gy, alteration of the *spermine synthase *gene, rather than *Phex*, is responsible for inner ear defects [44]. We should stress here that the inner ears of Hyp(Kbus) have normal histological features, and their characteristic bustling behavior is relatively slight compared with that of inner ear defect-bearing BUS mice [1]. Furthermore, the behavioral trait was almost nullified in F2 progeny in outcrosses with C57BL/6 mice, suggesting the Hyp(Kbus) behavior to be background-dependent. No clear association between defective *PHEX *and inner ear defects has been specified in humans, while some XLH patients have hearing impairment [45].

Hyp models have greatly contributed to our understanding of bone matrix mineralization and maintenance of the Pi balance. Aberration of the Phex-SIBLINGs system leads to remarkable elevation in FGF23 [46-49], and it is now evident that increased levels of FGF23, ASARM and MEPE account for various pathophysiological phenomena described earlier in XLH and Hyp animals. However, the implications and mechanisms of Hyp-induced elevation of FGF23 have remained unclear, because the main sites of FGF23 production are osteoblasts/osteocytes [8,9], and because Phex deficiency should result in diminished osteogenesis. Equally unclear have been the source and implications of increased serum ALP activity in Phex deficiency. In general, serum ALP activity and the number of ALP-positive osteoprogenitor cells in BMSC culture correlate with bone-forming ability and bone density [50]. In Hyp models, however, this is not the case, because cultured Hyp BMSCs exhibit reduced osteoblastogenesis and skeletogenesis [24-26]. Based on the present finding that isolated Hyp(Kbus) BMSCs exhibit significantly elevated ALP activity, we suggest that ALP-containing osteoblast-like cells in the bone marrow are the source of ALP increase in serum, further indicating that most of these ALP-positive BMSCs could be non-adherent or unable to survive in culture. It is necessary to specify the role and the fate of ALP-positive cells abundant in Hyp(Kbus) bone marrow.

Hyp models have another important contribution as research tools for therapeutic approaches. It appears that rescue of the Hyp phenotype can be accomplished by expression of the *PHEX *transgene over a long period of time under control of a bone-specific promoter [51], although in some reports there was only partial rescue by the transgene of Hyp abnormalities [52-54]. Recently, it has been noted that Hyp/klotho-/- knockout mice lack the hypophosphatemia and mineralization defects of Hyp, but with a shortened life span [55,56]. Hyp models can be expected to play a part in providing further clues to therapeutic manipulation of Pi balance-associated disorders.

## Conclusions

Histopathological and molecular genetic analyses here demonstrated a newly established dwarfism-like Kbus/Idr mouse line to be a novel Hyp model. The mutant could be important as a tool in further dissection and understanding of regulatory mechanisms of bone mineralization and Pi homeostasis, and in assessment of therapeutic aspects of human bone/Pi-associated disorders.

## Competing interests

The authors declare that they have no competing interests.

## Authors' contributions

KM of Mukogawa Women's Univ. carried out histopathological survey and prepared the manuscript. AH of Institute for Developmental Research established the Kbus strain and carried out cross experiments. KM and AY of RIKEN carried out X-ray irradiation experiments and molecular genetic study. KO, AK and TT of Hokkaido University carried out ALP and TRAP assays. These contributors are continuing analysis of Kbus abnormalities using a cell culture system. All authors read and approved the final manuscript.

## Supplementary Material

Additional file 1 **Figure S1. Comparisons of whole body skeletal preparations of KYF and Kbus mice**. The arizarin Red S/alcian blue staining method [42] was applied. Each bone of Kbus mice (right specimen in each panel) is shorter than the counterpart of KYF (left specimen), which is apparent in the long bones and bony segments of the tails. The skeletal abnormalities result in a shorter tail and dwarfism-like looks of Kbus mice. 1, 0-day-old. 2, 5-day-old. 3, 20-day-old. 4, 4-week-old. Click here for file

Additional file 2 **Figure S2. Histological examinations with Kbus and KYF femurs**. Femur bones from Kbus and KYF mice, 3-week-old (3 wks) and 8-week-old (8 wks), were fixed in Bouin's solution and decalcified with neutral 10% EDTA. 8-10 μm paraffin sections were cut, followed by Masson's trichrome stain (MT) or toluidine blue stain (TB). Note many sinuses existing in the cortical bones of Kbus adults, indicating a deranged Haversian system. The existence of thick growth plates in Kbus cartilages is also evident, which is one of the characteristic features of cartilage abnormalities. Click here for file

Additional file 3 **Table S1. Primer sets used for identifying the *Phex *transcripts and *Phex *exons 15-20. Table S2. Blood phosphorus levels of KYF and Kbus mice**. Click here for file

Additional file 4 **Figure S3. Dwarfism-like Kbus mice, originated from a breeding stock of KYF/MsIdr mice, are smaller than KYF mice at any age**. Each bar represents the mean of body weight values of at least 20 individuals. Compare the red bars (Kbus female) with the brown ones (KYF female), and the blue bars (Kbus male) with the grey ones (KYF male). Click here for file
