# Supplementary figures and images for: Kbus/Idr, a mutant mouse strain with skeletal abnormalities and hypophosphatemia: Identification as an allele of 'Hyp'
Source: J Biomed Sci. 2011 Aug 20;18(1):60. doi: 10.1186/1423-0127-18-60 (PMC3175157; doi:10.1186/1423-0127-18-60)

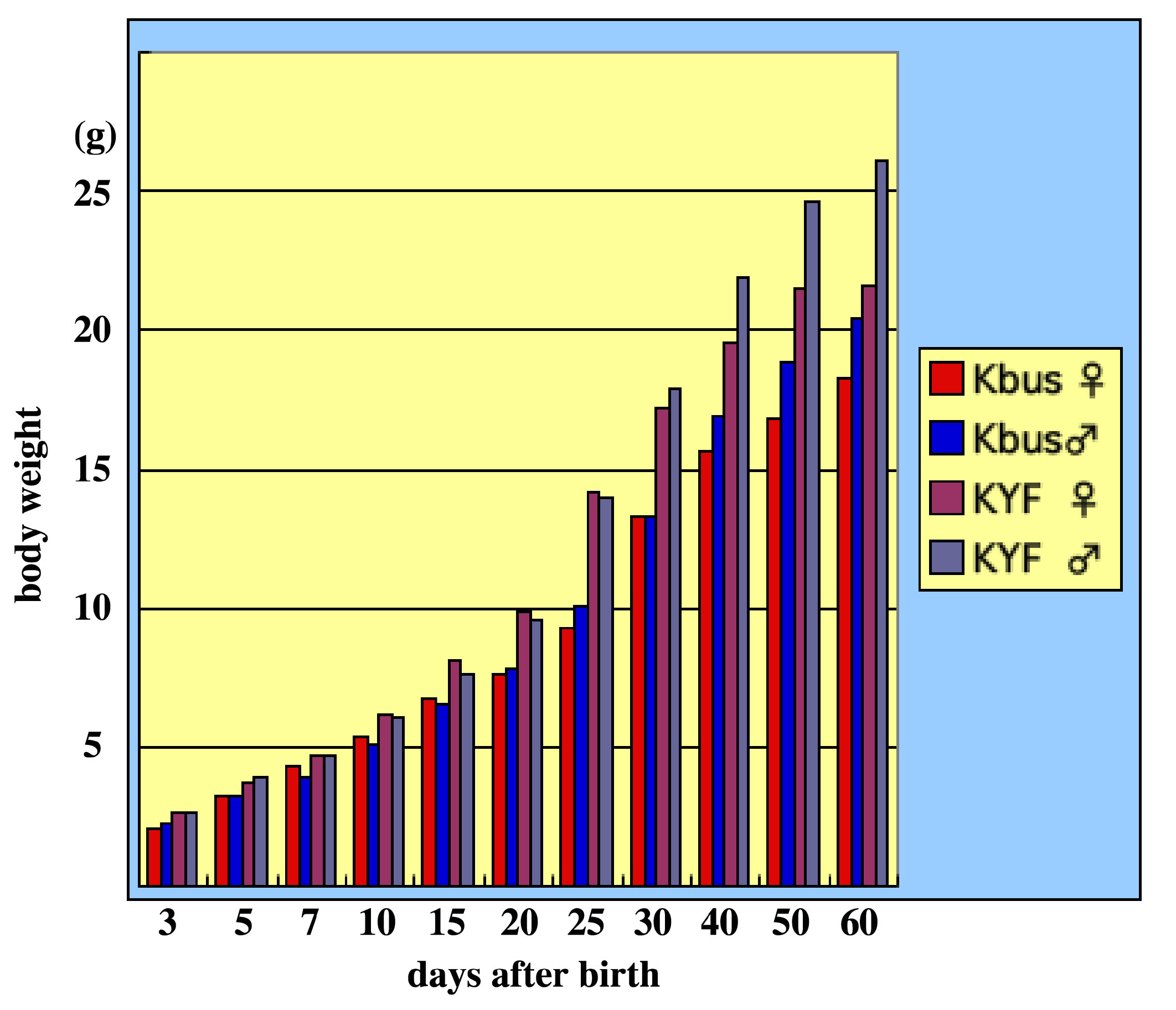

Supplement: Additional file 1 — Figure S1. Comparisons of whole body skeletal preparations of KYF and Kbus mice. The arizarin Red S/alcian blue staining method [42] was applied. Each bone of Kbus mice (right specimen in each panel) is shorter than the counterpart of KYF (left specimen), which is apparent in the long bones and bony segments of the tails. The skeletal abnormalities result in a shorter tail and dwarfism-like looks of Kbus mice. 1, 0-day-old. 2, 5-day-old. 3, 20-day-old. 4, 4-week-old. [file 1423-0127-18-60-S1.JPEG]

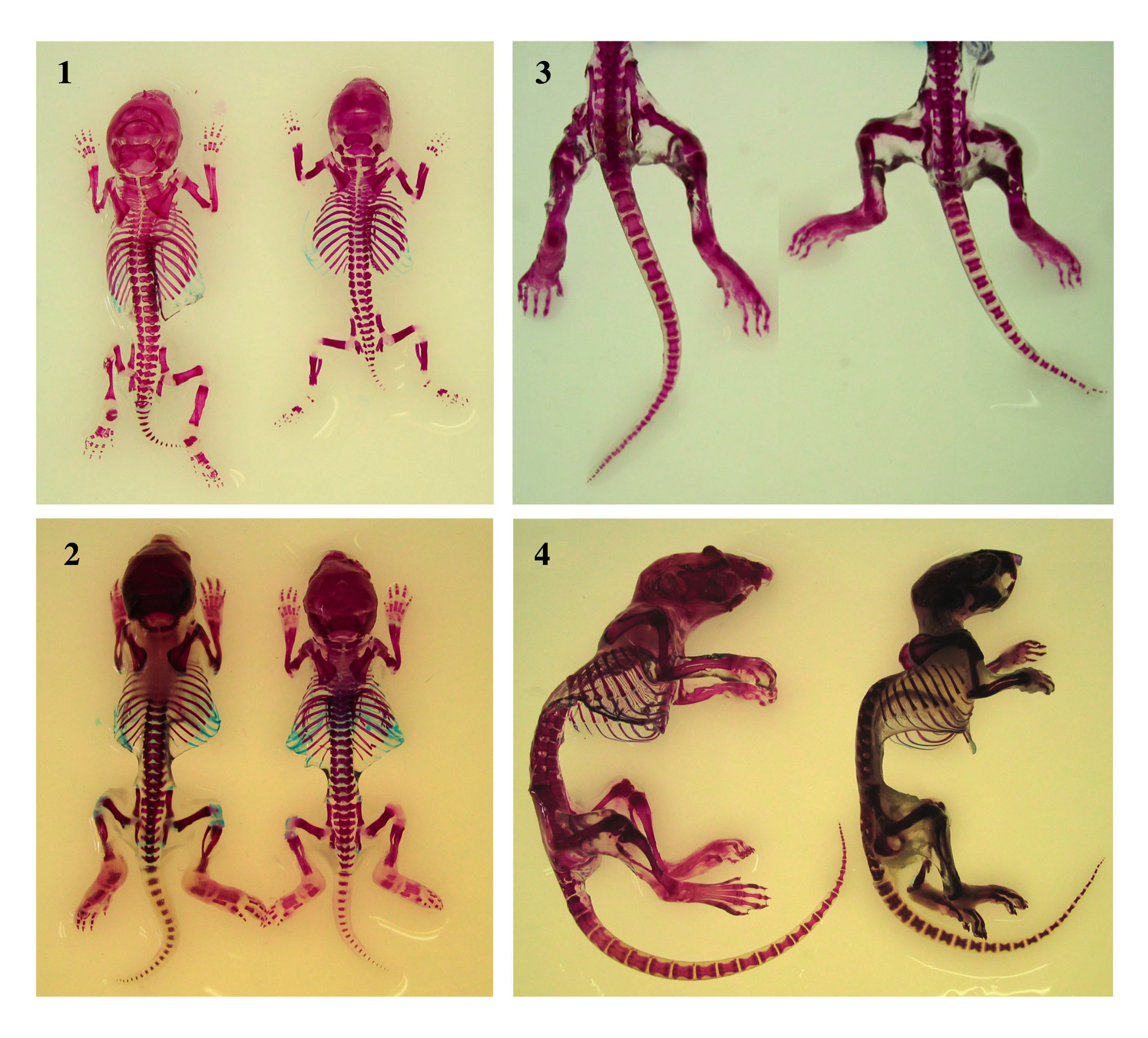

Supplement: Additional file 2 — Figure S2. Histological examinations with Kbus and KYF femurs. Femur bones from Kbus and KYF mice, 3-week-old (3 wks) and 8-week-old (8 wks), were fixed in Bouin's solution and decalcified with neutral 10% EDTA. 8-10 μm paraffin sections were cut, followed by Masson's trichrome stain (MT) or toluidine blue stain (TB). Note many sinuses existing in the cortical bones of Kbus adults, indicating a deranged Haversian system. The existence of thick growth plates in Kbus cartilages is also evident, which is one of the characteristic features of cartilage abnormalities. [file 1423-0127-18-60-S2.JPEG]

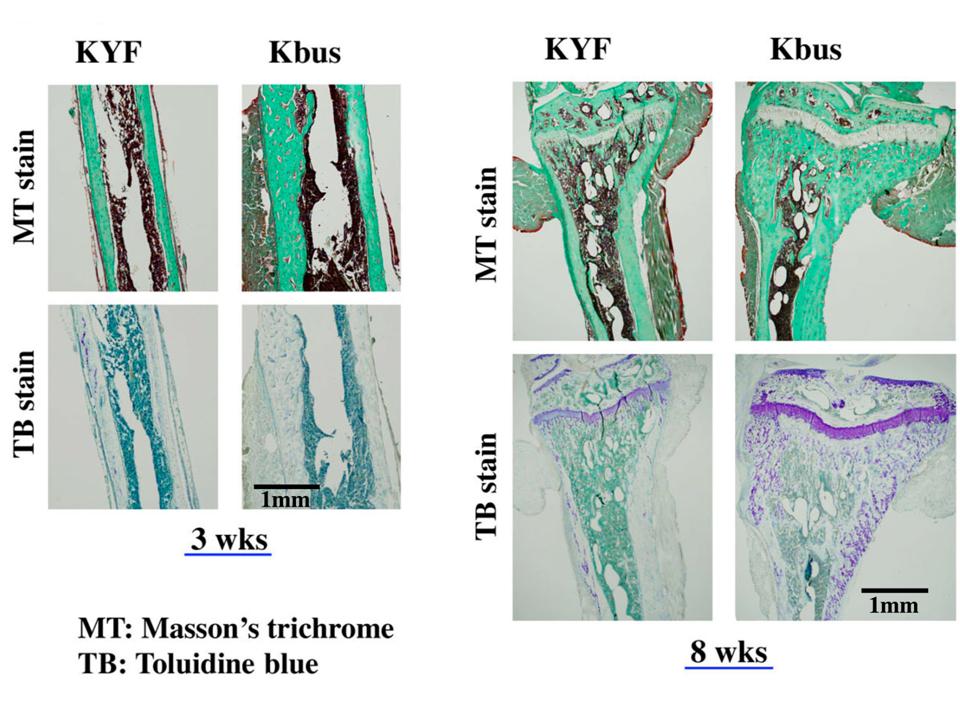

Supplement: Additional file 4 — Figure S3. Dwarfism-like Kbus mice, originated from a breeding stock of KYF/MsIdr mice, are smaller than KYF mice at any age. Each bar represents the mean of body weight values of at least 20 individuals. Compare the red bars (Kbus female) with the brown ones (KYF female), and the blue bars (Kbus male) with the grey ones (KYF male). [file 1423-0127-18-60-S4.JPEG]
