# Supplementary material for: Kbus/Idr, a mutant mouse strain with skeletal abnormalities and hypophosphatemia: Identification as an allele of 'Hyp'
Source: J Biomed Sci. 2011 Aug 20;18(1):60. doi: 10.1186/1423-0127-18-60 (PMC3175157; doi:10.1186/1423-0127-18-60)
Supplement: Additional file 3 — Table S1. Primer sets used for identifying the Phex transcripts and Phex exons 15-20. Table S2. Blood phosphorus levels of KYF and Kbus mice. [file 1423-0127-18-60-S3.DOC]

**Table S1. Primer sets used for identifying the *Phex* transcripts and *Phex* exons 15-20**

exon nt. number Product size

PHEXF1 　5’TTCTAAGTCTTCTCTCCAGCC3’ 1 nt -27〜 nt -7 460bp

PHEXR1 　5’CATGCAGGATGAGTAGAGGAT3’ 4 nt 429〜 nt 409

PHEXF2　 5’GTTGACCTCAAGTTGAAGGCA3’ 2-3 nt 331〜 nt 351 494bp

PHEXR2　 5’AGCACCGACTTCATGTCATGT3’ 7 nt 824〜 nt 804

PHEXF3 　5’CAAATCTTATCGGGATGCCCT3’ 6-7 nt 726〜 nt 746 525bp

PHEXR3　 5’CATATGGGAGTGCACTCTCAA3’ 11 nt 1249〜 nt 1229

PHEXF4　 5’AGAATTCCAAACCTCAGCAGG3’ 10 nt 1120〜 nt 1140 515bp

PHEXR4　 5’GGTAGATGCACTGTAAAAGGC3’ 15 nt 1635〜 nt 1615

PHEXF5　 5’CCCGCAAGTATTTAGCACAGT3’ 14 nt 1526〜 nt 1546 515bp

PHEXR5　 5’TGTTGGTGAATGTGATGCCTG3’ 20 nt 2041〜 nt 2021

PHEXF6　 5’ATTGCTGATAATGGGGGTCTG3’ 19 nt 1930〜 nt 1950 370bp

PHEXR6　 5’CTACTGGCATTCCTCAGCTAT3’ 22 nt 2299〜 nt 2279

Exon15F: 5’GATCAATGCAATCCCCCTACACA3’ (intron 14) 140bp

Exon15R: 5’TGATGGCTACCTTCTGGCATTTG3’ (intron 15)

Exon16F: 5’GCTAACTTGTCAAGGTCAGGTTT3’ (intron 15) 170bp

Exon16R: 5’GGAACCAGAGTCTGGATATTGAT3’ (intron 16)

Exon17F: 5’CCTGATCACCAACTGGAATAGTT3’ (intron 16) 500bp

Exon17R: 5’GCTCATATAAGGTCACTGTGACA3’ (intron 17)

Exon18F: 5’ATGAAGAAAAATGGCCCACAAGG3’ (intron 17) 300bp

Exon18R: 5’GGCTAGTTTGGAAAGTATGTCCA3’ (intron 18)

Exon19F: 5’CACCTATGCAGGCTTTCATAAGT3’ (intron 18) 170bp

Exon19R: 5’TGCTGAATGGTAGTTGACTGTGA3’ (intron 19)

Exon20F: 5’CCCATGGCAGGAATATGTGTTTA3’ (intron 19) 240bp

Exon20R: 5’ATACATTTTCCCCTGTGATGGTC3’ (intron 20)

**Table S2. Blood phosphorus levels of KYF and Kbus mice.**

Pi (μmol/ml of blood)

male

KYF 1.73 ± 0.09

Kbus 0.97 ± 0.08*

female

KYF 1.80 ± 0.11

Kbus *homo* 1.00 ± 0.09*

*hetero* 0.99 ± 0.09*

Values are presented as the means ± SEM (n=3).

Asterisks indicate *P* < 0.01 against the KYF values.
